# Endoscopic sinus surgery improve quality of life and decrease absenteeism in patients with chronic rhinosinusitis - a multi-centre study

**DOI:** 10.1186/2045-7022-5-S4-O6

**Published:** 2015-06-26

**Authors:** Pernilla Sahlstrand-Johnson, Bodil Ohlsson, Marianne Ahlner-Elmqvist

**Affiliations:** 1 Skåne University Hospital, Dep of Otorhinolaryngology, 20502, Malmö, Sweden; 2 Skåne University Hospital, Department of Clinical Sciences, Malmö, Sweden; 3 Lund University, Department of Health Sciences, Lund, Sweden

## Background

We have previously reported that chronic rhinosinusitis (CRS) has a significant impact on health-related quality of life (HRQOL). In this study we aimed to analyse whether sino-nasal symptom scoring can be used to predict the postoperative outcome measured by HRQOL. Secondly, we wanted to investigate whether absenteeism caused by sinus symptoms decrease after endoscopic sinus surgery (ESS). We also wanted to assess whether the duration of sinus disease had an effect on the outcome of ESS.

## Methods

Two hundred and seven patients with CRS with and without nasal polyps (NP) admitted for ESS from ten hospitals were enrolled. EPOS2012 definitions of CRS and NP were used. The patients completed the short-form 36-item questionnaire (SF-36), the 22 Sinonasal Outcome Test (SNOT-22) and a total Visual Analogue Scale (VAS) regarding rhinosinusitis symptoms preoperatively, 6 and 12 months after surgery.

## Results

Of the 207 patients, 135 were diagnosed with CRS+NP and 72 CRS-NP. Sixty-nine percent of the population had anterior ethmoidectomy, 36% posterior ethmoidectomy and 24% surgery of the frontal sinus recess. The quality of life scores measured by SF-36 improved in all eight domains but one after surgery. The total SNOT-22 score decreased significantly from 51.8 to 33.0 at 6 months postop (p<0.0001) and stayed at this level 12 months after surgery. There was no statistically significant difference in total SNOT-22 scoring between patients with and without NP, but in four of the SNOT-22 questions there were differences. The total VAS scores dimidiated from 67 to 33 postoperatively. Sick-leave due to rhinosinusitis dropped from 8-14 days to 1-7 days 12 months after ESS. The questions on “Lack of a good night’s sleep” and “Frustrated/restless/irritable” in SNOT-22 were predictive (p=0.001) for the postoperative total SNOT-22 score, when performing a backwards stepwise regression analysis. Those patients who had had a history of less than 12 months of sinus disease scored best postoperatively measured by SNOT-22.

## Conclusion

Quality of life improved significantly after ESS measured by SF-36, SNOT-22 and VAS. Scoring of two of the questions in SNOT-22 proved to be significantly predictive for the postoperative outcome. Patients with CRS±NP reported less absenteeism due to sino-nasal disease 12 months postoperatively. The patients with shorter sinus disease duration seem to gain more by sinus surgery, which is something we need to look further at.

